# The Sale of Food and Drugs Act, 1899

**Published:** 1900-01-20

**Authors:** 


### THE SALE OF FOOD AND DRUGS ACT, 1899.

THIS Act came into force on January 1st, 1900. Its various provisions, which are contained in twenty-seven sections, evoked considerable discussion when the measure was being piloted through the House of Commons by Mr. Long. Circulars have already been issued by the Board of Agriculture, drawing attention to the provisions of the Act, and to the manner in which it is to be administered. The short effect of the more important sections is as follows:—According to Section 1, margarine or margarine cheese, adulterated or impoverished butter, condensed, separated, or skimmed milk, must not be imported into the United Kingdom unless the packages or cans containing the same are conspicuously marked in such a way as to show a purchaser the exact nature of the substance which he is buying. The same rule may be applied to any article of food by Order in Council. Infringement of the provisions of this section involves a fine of not exceeding £20 for the first offence, and not exceeding £50 for the second, and for any subsequent offence not exceeding £100. The word "importer" includes any person who, whether as owner, consignor, or consignee, agent, or broker, is in possession of, or in anywise entitled to, the custody or control of the article. An article of food is deemed to be adulterated or impoverished if it has been mixed with any other substance, or if any part of it has been abstracted so as in either case to affect injuriously its quality or substance or nature. Mere preservative or colouring matter is not to be regarded as adulteration. Margarine may, therefore, still be coloured so as to look like butter. Section 2 gives power to the Local Government Board, on behalf of the consumer, and to the Board of Agriculture, in the interests of agriculture, to obtain and analyse samples of any article of food. The result of the analysis is to be communicated to the local authority. By Section 3 it is declared to be the duty of the local authority to appoint a public analyst, who must furnish such proof of competency as may from time to time be required by regulations framed by the Local Government Board. It also gives power for either Board to act in default of the local authority. "Even if nothing but this clause had survived," said Sir William Foster, in Parliament, "there would have been a great improvement effected by the Act."

Section 4 gives power to the Board of Agriculture to declare, after due inquiry, what proportion of extraneous matter shall be sufficient to raise a presumption that the article in question is not genuine. Section 5 extends the application of the Margarine Act, 1887, to margarine cheese, while Section 6 provides that packages of margarine or margarine cheese must be marked not only on a label or ticket, but on the actual package itself. Section 7 contains provisions as to manufacturers of and dealers in margarine and margarine cheese. They must keep a register showing the quantity and destination of each consignment sent out. Heavy penalties are imposed for neglect to comply with this section.

By Section 8 it is declared to be unlawful to sell margarine the fat of which contains more than 10 per cent. of butter fat A person charged with so doing may, however, raise the defence that he bought the article with a written warranty. This section was strenuously opposed by Sir Charles Cameron. It was passed with the object of preventing the sale of any mixture which could be passed off as butter. Section 9 provides that every person selling milk or cream in a public place shall have his name and address conspicuously inscribed on the vehicle or receptacle upon or in which he carries the milk. Section 11 provides that every tin or receptacle containing condensed, separated, or skimmed milk shall be provided with a conspicuous [illegible]bel to that effect. Mr. Long refused to sanction the addition of any words to the effect that such milk should not be given to infants.

As the penalties heretofore imposed for breaches of the Sale of Food and Drugs Act, 1875, have been found to be too light, it is now provided by Section 17 of the Act under review that where any person has been found guilty of an offence under the previous Act which would expose him to a fine not exceeding £20, a second offence may be met with a fine not exceeding £50, and any subsequent offence to a fine not exceeding £100. Moreover, the courts of summary jurisdiction are now empowered to sentence the offender to imprisonment for a period not exceeding three months, in any case where he may be liable to a fine not exceeding £50.

In order to avail himself of the defence that the goods were purchased under warranty or invoice, the defendant must in future, by virtue of Section 20, send to the purchaser (1) a copy of the warranty or invoice; (2) a written notice stating that he intends to rely upon it, and specifying the name and address of the person from whom he received it. Persons giving false warranties or invoices are subjected to very severe penalties.

